# Case Report: Allelic and biallelic variants in *coagulation factor XI* cause factor XI deficiency

**DOI:** 10.3389/fcvm.2024.1461899

**Published:** 2024-11-13

**Authors:** Chen Liang, Jie-Yuan Jin, Hai-Hong Shi, Hao-Xian Li, Lin-Lin Chen, Yang-Hui Zhang, Qin Wang, Qiu-Li Li, Rui-Man Li

**Affiliations:** ^1^ Department of Clinical Research, The First Affiliated Hospital of Jinan University, Guangzhou, China; ^2^ Center for Medical Genetics, Jiangmen Maternal & Child Health Care Hospital, Jiangmen, China; ^3^ School of Life Sciences, Central South University, Changsha, China; ^4^ Department of Pediatrics, Jiangmen Maternal & Child Health Care Hospital, Jiangmen, China; ^5^ Center of Reproductive Medicine, Jiangmen Maternal & Child Health Care Hospital, Jiangmen, China; ^6^ Department of Nephrology, Xiangya Hospital, Central South University, Changsha, China; ^7^ Department of Obstetrics and Gynecology, The First Affiliated Hospital of Jinan University, Guangzhou, China

**Keywords:** Factor XI deficiency, hemophilia, *coagulation factor XI*, variant characteristic, missense variant

## Abstract

Factor XI deficiency is a rare inherited coagulation disorder with an estimated prevalence of affecting 1 in 1 million. It is characterized by mild and variable bleeding phenotypes, including bruises, nosebleeds, hematuria, and postpartum hemorrhage. It can be caused by either allelic or biallelic variants in *coagulation factor XI* (*F11*). Coagulation factor XI is a glycoprotein that circulates in plasma as a non-covalent complex with high-molecular-weight kininogen. It is converted to an active protease, coagulation factor XIa, which participates in blood coagulation as a catalyst. In this study, we recruited a family with Factor XI deficiency and identified two *F11* variants using whole-exome sequencing. One (NM_000128.4: c.841C>T, p.Q281X) was a known variant, and the other (NM_000128.4: c.1832T>G, p.V611G) had not been reported. In addition, we compiled the characteristics of known missense variants in *coagulation factor XI*. Our findings enriched the variant spectrum of Factor XI deficiency and contributed to the genetic counseling and molecular diagnostics of Factor XI deficiency.

## Introduction

Hemophilia is a group of classical coagulation disorders that can result in excessive bleeding, requiring intervention to restore hemostasis (1). Hemophilia can be categorized into hemophilia A (Factor VIII deficiency, OMIM_306700), resulting from *coagulation factor VIII* (*F8*) variants; hemophilia B (Factor IX deficiency, OMIM_306900), caused by *coagulation factor IX* (*F9*) variants; and Factor XI deficiency (OMIM_612416), associated with *coagulation factor XI* (*F11*) variants (2–4). Compared with Factor VIII and IX deficiencies, Factor XI deficiency is notably rare, with a prevalence of approximately one in one million (1). It typically presents with mild and variable bleeding phenotypes, such as bruises, epistaxis, hematuria, and postpartum hemorrhage, and severe spontaneous bleeding is infrequent (5). This disease is usually found in the following conditions: trauma or surgery-induced excessive bleeding, routine pre-surgical laboratory evaluation, and genetic screening of a probands’ family members (6).

Factor XI deficiency is associated with biallelic variants of *F11*, whereas certain heterozygous variants in *F11* can also result in this disorder, with milder phenotypes and incomplete penetrance (7). Coagulation factor XI is a glycoprotein that circulates in plasma as a non-covalent complex with high-molecular-weight kininogen. It is converted to an active protease, coagulation factor XIa, in the presence of calcium ions, participating in blood coagulation as a catalyst (8, 9). Options for treatment with coagulation factor XI include fresh frozen plasma, coagulation factor XI concentrates, and low-dose recombinant coagulation factor VIIa, with antifibrinolytic agents as an adjunctive therapy (10).

In this study, we reported a family with Factor XI deficiency caused by heterozygous variants or compound heterozygous variants in *F11* (NM_000128.4: c.841C>T, p.Q281X; c.1832T>G, p.V611G). Our identification enriched the variant spectrum of Factor XI deficiency. We compiled the features of known missense variants in *coagulation factor XI* and provided evidence to support genetic counseling and molecular diagnostics in Factor XI deficiency.

## Materials and methods

### Subjects

This study received ethical approval from the Review Board of Jiangmen Maternal & Child Health Care Hospital [No. 112 (2022)]. A pregnant woman diagnosed with Factor XI deficiency and her family members were recruited. All of the participants (Han ethnic group) provided written informed consent for their participation in this study and the publication of related data. This study was conducted in accordance with the ethical standards of the 1964 Declaration of Helsinki and its subsequent amendments.

### Whole-exome sequencing

Peripheral blood samples of subjects were collected, and their genomic DNAs were extracted. Berry Genomics Company Limited (Beijing, China) performed whole-exome sequencing (WES) of the proband following protocols as previously described (11). Given that the proband was diagnosed with Factor XI deficiency, we specifically analyzed variants in *F8*, *F9*, and *F11*. Variant detection rates were annotated according to GnomAD (http://gnomad.broadinstitule.org) and the Chinese Millionome Database (CMDB; http://cmdb.bgi.com/cmdb/). MutationTaster (http://www.mutationtaster.org), SIFT (http://provean.jcvi.org/index.php), PolyPhen-2 (http://genetics.bwh.harvard.edu/pph2), Combined Annotation Dependent Depletion (CADD) (https://cadd.gs.washington.edu/snv), and MetaDome (https://stuart.radboudumc.nl/metadome/dashboard) were utilized to predict the pathogenicity of the variants. The pathogenicity classification of the variants was confirmed in adherence with the standards and guidelines of the American College of Medical Genetics and Genomics (ACMG) (12].

### Sanger sequencing

The variants in all of the participants were verified using Sanger sequencing. Gene sequencing of *F11* was obtained from the National Center for Biotechnology Information (NCBI) (https://www.ncbi.nlm.nih.gov/gene/2160). Primer pairs (F11-841f: 5′-GCTAGCATGAGCTGACTTTACT-3′, F11-841r: 5′-TCTCAGCCAGAATGCAGAAC-3′; F11-1832f: 5′-GAAGCGTCTGAGTTGATCTGT-3′, F11-1832r: 5′-TTCAGCGTGTTACTGTGGAG-3′) were synthesized by Sangon Biotech Company Limited (Shanghai, China).

### Three-dimensional protein modeling, hydrophobicity analysis, and variant compilation

The three-dimensional models of coagulation factor XI were downloaded from the AlphaFold database (https://alphafold.ebi.ac.uk/entry/P03951), and PyMOL was used to establish the mutant protein models. The hydrophobicity of coagulation factor XI and its mutant proteins was predicted using Expasy (https://web.expasy.org/protscale/).

Missense variants were documented based on the Human Gene Mutation Database (HGMD) (https://www.hgmd.cf.ac.uk/ac/gene.php?gene=F11) and the PubMed database (https://pubmed.ncbi.nlm.nih.gov/?term=F11+mutation&size=200). Compared with polar amino acids, methionine and glycine are hydrophobic and they have higher hydrophilicity than other non-polar amino acids. The disulfide bond structures of the variants were determined by referring to the AlphaFold database.

## Results

The proband (II: 2; Figure 1A) was a 31-year-old woman at 29 weeks of gestation (first pregnancy). According to her self-statement, 4 years ago, she was diagnosed with Factor XI deficiency [her activated partial thromboplastin time was 106.0 s (normal reference range: 28–38 s) and coagulation factor XI level was below 1%] during a preoperative examination of uterine fibroids. Except for a plasma transfusion after the operation (conducted without major bleeding), she did not receive any other treatment. Recently, she had observed an ecchymosis had appeared on her right hand again. She requested that our department assess the risk and severity of Factor XI deficiency in her baby.

**Figure 1:**
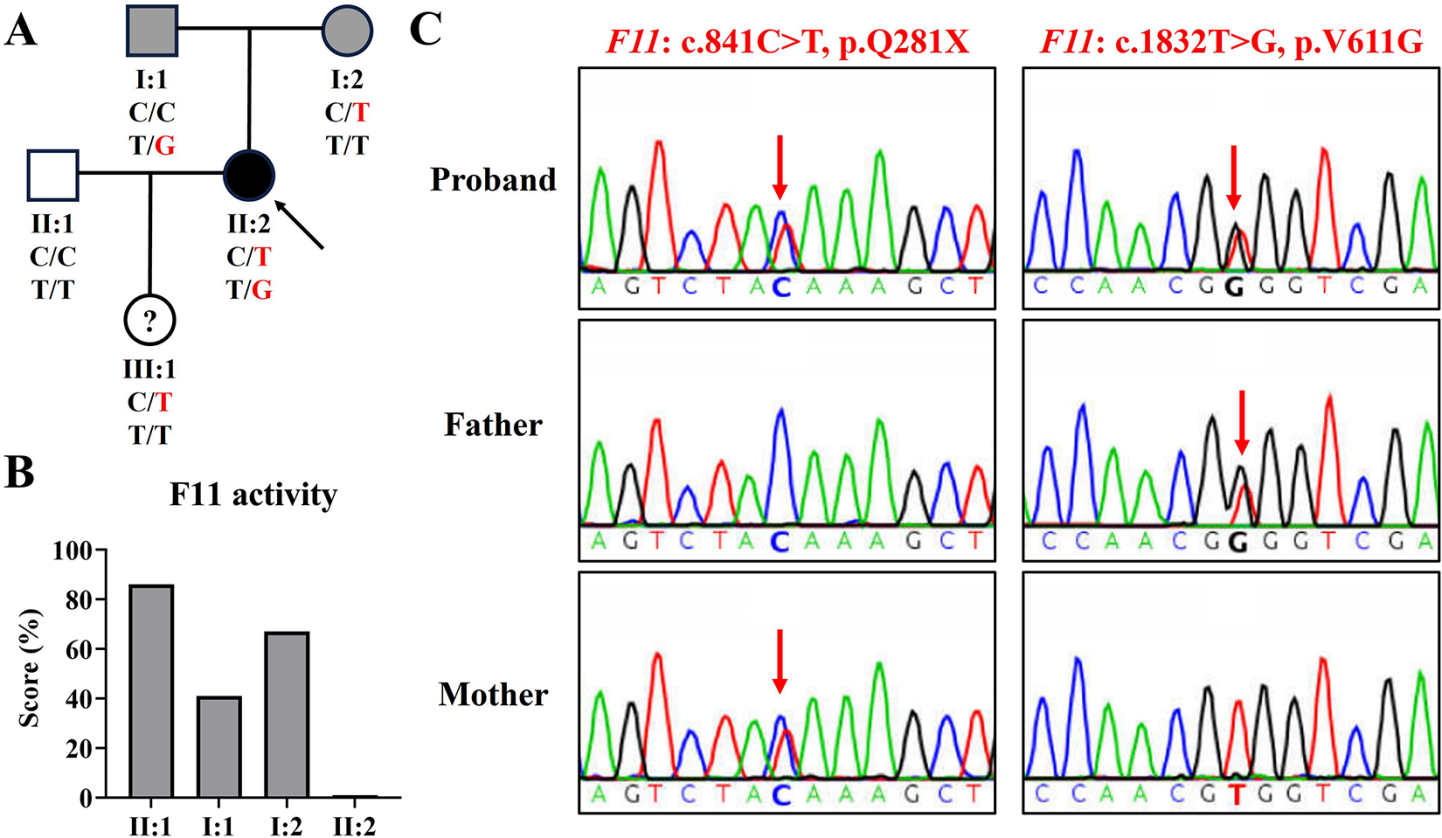
The family genogram, *F11* activity, and sanger sequencing of the family. **(A)** The family pedigree of the family. The black symbol represents the affected member, gray symbols represent mild phenotype, “?” represents phenotype unknown, the arrow indicates the proband, and red text represents the variant. **(B)** The activity levels of coagulation factor IX in the family. **(C)** Sequence chromatograms of the proband and her parents. Red arrows indicate variant sites.

At this visit to our department, a coagulation factor XI activity test revealed that her coagulation factor XI activity was less than 1% (normal reference range: 72%–130%), her husband's (II: 1) was 86%, her father's (I: 1) was 41%, and her mother's (I: 2) was 67% (Figure 1B). This indicated that the proband was seriously deficient in coagulation factor XI, and her parents also had mild deficiencies. In addition, her platelet count was 232 × 10^9^ L (normal reference range: 100–300 × 10^9^ L) and her plasma fibrinogen concentration was 2.17 g/L (normal reference range: 2.00–4.00 g/L).

Using WES, we identified two *F11* variants (NM_000128.4: c.841C>T, p.Q281X; c.1832T>G, p.V611G) in the proband (Table 1). Variant c.1832T>G, p.V611G was novel and variant c.841C>T, p.Q281X has been reported several times (5, 13). Sanger sequencing confirmed that variant c.841C>T was inherited from her mother and variant c.1832T>G was derived from her father (Figure 1C). Her husband tested negative for both variants.

**Table 1:** The information and pathogenicity classification of the variants identified in the proband.

Gene	Variant	Pathogenicity prediction	GnomAD	CMDB	OMIM clinical phenotype	ACMG classification
*F11*	NM_000128.4: c.841C>T, p.Q281X	MutationTaster: D SIFT: PolyPhen-2: CADD: 36	0.00009	0.00110	AD, Factor XI deficiency, autosomal dominant; AR, Factor XI deficiency, autosomal recessive	Likely pathogenic (PVS1, PS1, PM3)
	NM_000128.4: c.1832T>G, p.V611G	MutationTaster: D SIFT: D PolyPhen-2: D CADD: 26.9	—	—		Likely pathogenic (PM2, PM3, PM5, PP3)

GnomAD, the Genome Aggregation Database; CMDB, Chinese Millionome Database; OMIM, Online Mendelian Inheritance in Man; ACMG, American College of Medical Genetics; D, disease-causing; —, non-existence data; AD, autosomal dominant; AR, autosomal recessive; PVS, very strong pathogenicity; PS, strong pathogenicity; PM, moderate pathogenicity; PP, supporting pathogenicity.

**Figure 2:**
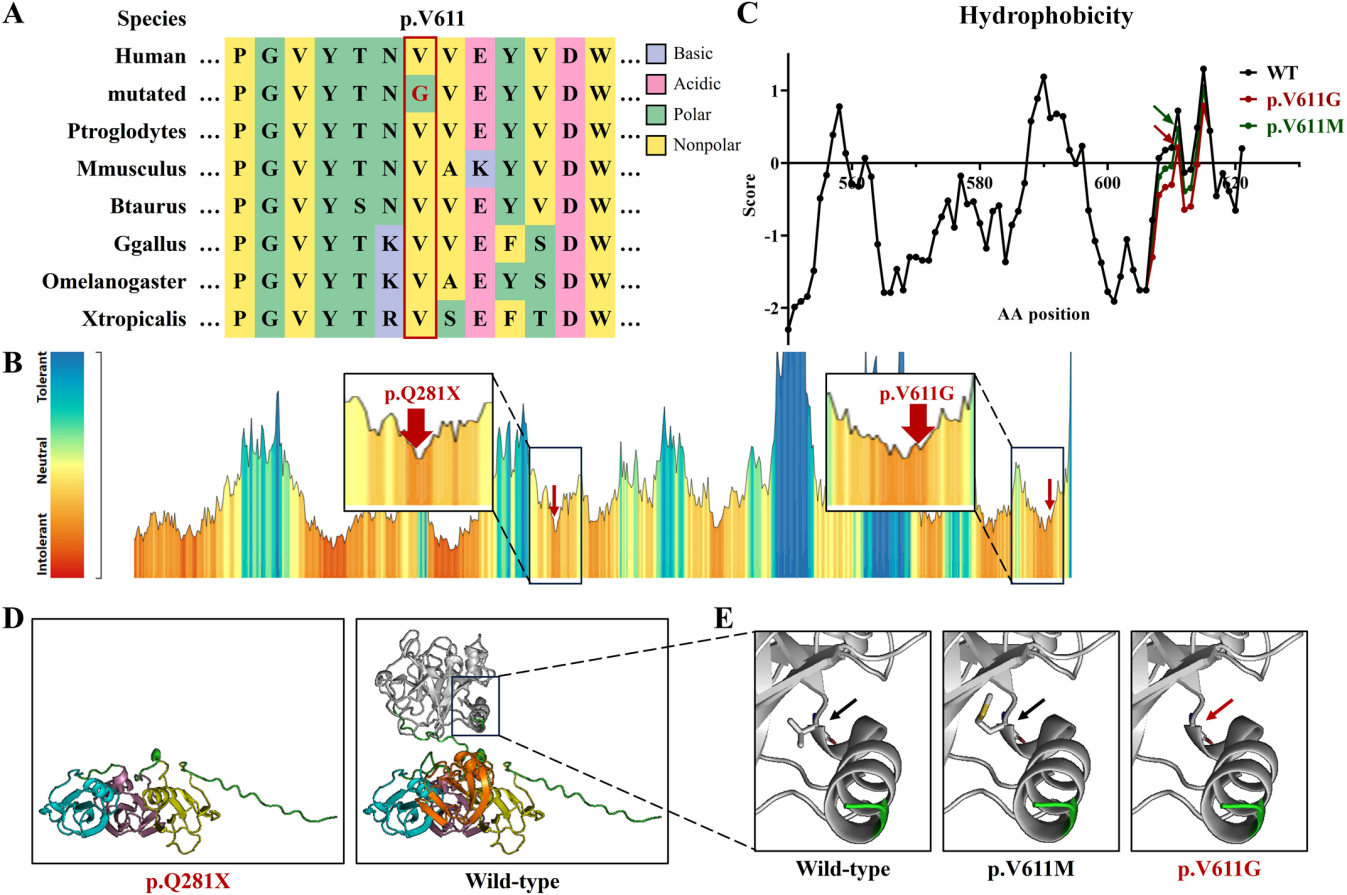
The bioinformatics analysis of the *F11* variants in this study. **(A)** The peptide sequences surrounding the mutated residues (p.V611G) with multiple interspecies alignments. Purple highlights basic amino acids, pink highlights acidic amino acids, green highlights polar amino acids, yellow highlights non-polar amino acids, and red text represents variant p.V611G from this study. **(B)** The intolerant prediction for functional genetic variations of coagulation factor IX. Red arrows indicate the variants in this study. **(C)** The hydrophobicity prediction of coagulation factor IX fragments with wild type (black), p.V611M (green), and p.V611G (red). **(D)** Protein models of coagulation factor IX with wild type, p.Q281X, p.V611M, and p.V611G. Yellow represents the Apple 1 domain, purple represents the Apple 2 domain, blue represents the Apple 3 domain, orange represents the Apple 4 domain, white represents the Protease domain, and red text represents the variants in this study.

These two *F11* variants were respectively classified as “Pathogenic” and “Likely pathogenic” in adherence with ACMG standards (Table 1). Null variants are one of the known pathogenic mechanisms of Factor XI deficiency, and variant c.841C>T was a nonsense variant (PVS1). This variant had been previously reported (PS1). The two *F11* variants were confirmed to be inherited from the patient’s parents (PM3). Variant c.1832T>G was absent from controls in the GnomAD and CMDB (PM2). However, variant p.V611M has been reported, and our variant, c.1832T>G, p.V611G, triggered a change in different residues at the same amino acid site (PM5) (14). p.V611 was highly conserved across evolution and was predicted by MetaDome to be intolerant to functional genetic variation (Figures 2A,B); predictions from MutationTaster, SIFT, PolyPhen-2, and CADD further supported the deleterious impact of variant c.1832T>G (PP3).

The hydrophobicity analysis showed that variant p.V611G reduced hydrophobicity at the variant site and its surrounding regions (Figure 2C). Three-dimensional protein modeling showed that variant p.Q281X produced a truncated protein and that variant p.V611G was positioned in an α-helix, without obvious structural changes (Figure 2D).

A patient follow-up showed that a cesarean section had been performed at 38 + 2 weeks of gestation due to the left occiput transverse (LOT) position of the fetus. The maternal blood loss was 630 ml within 2 h after delivery and 880 ml within 24 h, and was treated by the transfusion of two units of red blood cell (RBC) concentrates. The proband reported that she was in good condition and her baby is a girl (III: 1). Sanger sequencing showed that the baby harbored variant c.841C>T, p.Q281X, but her coagulation factor XI activity was not tested.

## Discussion

Factor XI deficiency is primarily caused by *F11* variants and is inherited in autosomal recessive or autosomal dominant patterns (7). Genetic screening is used to identify its etiologies and carry out molecular diagnosis. However, Sanger sequencing is only used to screen for variants in specific sequences, and there is a possibility that *F11* variants are not detected, for example, when checking whether a disease is triggered by copy number variations (CNVs) or other gene variants. In contrast, WES can screen for variants in the exons of all genes, rather than a certain gene, which is a more rigorous analysis strategy. Furthermore, WES can also be used to analyze CNVs, although this is not a gold-standard strategy. For these reasons, the proband chose WES, and Sanger sequencing was performed to detect the identified variants in all her family members. She harbored compound heterozygous variants in *F11* (NM_000128.4: c.841C>T, p.Q281X; c.1832T>G, p.V611G), resulting in severe Factor XI deficiency, while each parent carried one *F11* variant respectively and had a mild deficiency. Typically, homozygous variant carriers exhibit extremely low coagulation factor XI activity (0%–20%), and heterozygous variant carriers display partial deficiency (30%–70%) (15, 16). Our results align with these observations. The individual variants led to coagulation factor XI activity levels of 67% and 41%, respectively, while their compound heterozygous variants resulted in only 1% activity. In addition, we recommended that the proband be careful not to injure the fetus in the womb and talk to her attending doctor about her disease in advance to prepare for the possibility of needing a blood transfusion during her delivery. In fact, she did receive the transfusion of RBC concentrates in her cesarean section. The baby (III: 1) carried the variant c.841C>T, p.Q281X, but it was not tested whether she had a mild Factor XI deficiency.

**Figure 3:**
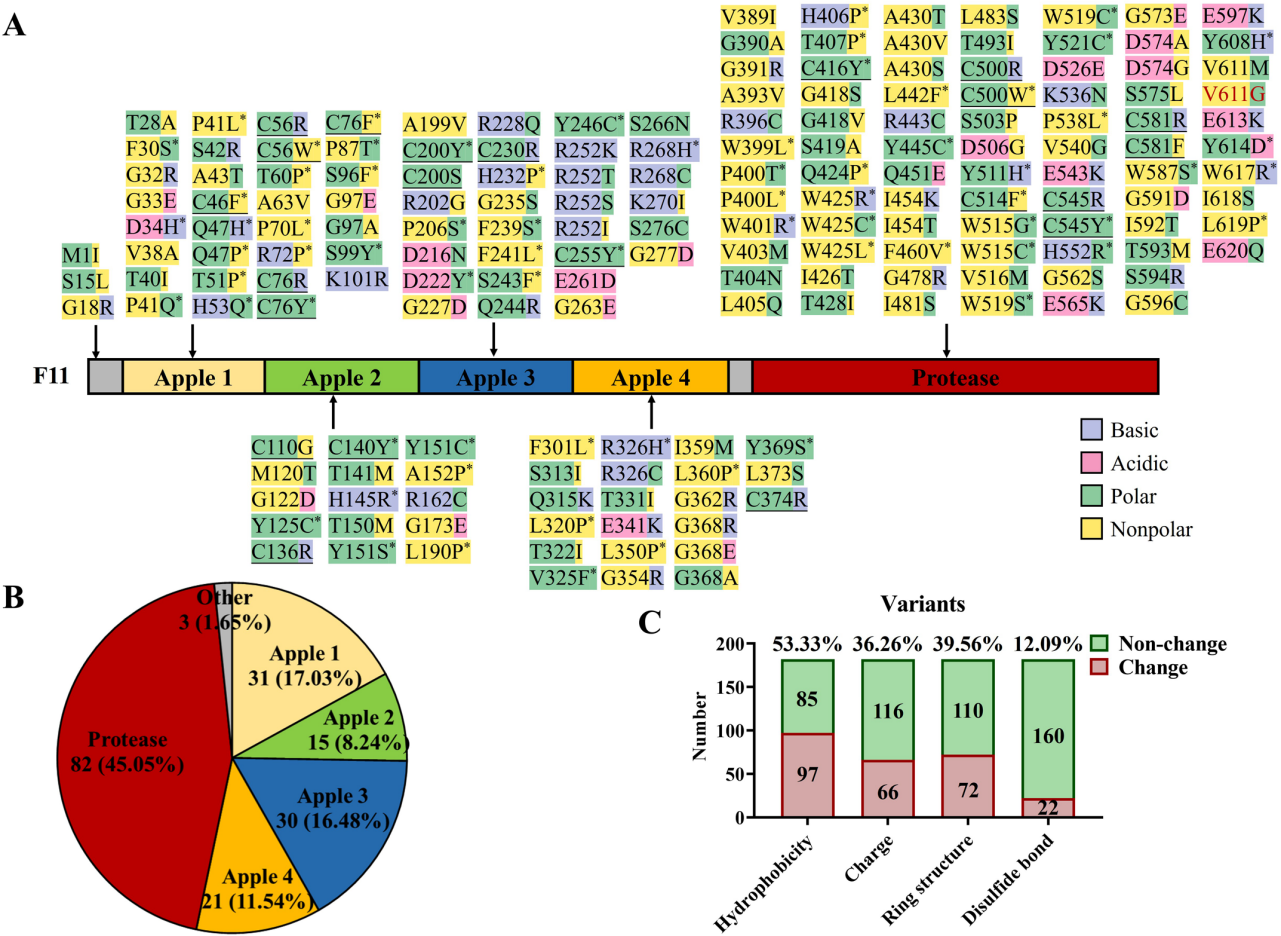
Known missense variants in *F11* reported in the literature and this report. **(A)** Protein structure domains and missense variant collection of coagulation factor IX. Purple highlights basic amino acids, pink highlights acidic amino acids, green highlights polar amino acids, yellow highlights non-polar amino acids, “*” represents the disappearance or acquisition of ring structure in residues, underline represents the destruction of disulfide bonds, and red text represent a variant in this study. **(B)** The proportion of *F11* missense variants in different domains. **(C)** A general illustration of the effects caused by *F11* missense variants.

Coagulation factor XI consists of four N-terminal domains, namely, plasminogen module (PAN)/Apple domains (Apple 1–4), and a serine proteases/trypsin domain (Protease) (9, 17). Apple domains can directly bind to kininogen, coagulation factor XIIa, platelets, coagulation factor IX, and heparin (9, 18, 19). Variant p.V611G is situated in the Protease domain, and a known variant, p.V611M, occurs in the same amino acid site (Figure 3A) (14). They both augment the hydrophilicity of the mutant region, without the destruction of the α-helix, but the increase in hydrophilicity due to our variant was greater than that of p.V611M (Figures 2C,D). The impacts triggered by p.V611G and p.V611M should be verified *in vitro* and/or *in vivo*.

Variant p.Q281X is positioned in the tail end of the Apple 3 domain, causing a loss of the Apple 4 domain and Protease domain (Figure 2D). According to data from the CMDB, the frequency of this variant in the Chinese population is 1.1 × 10^−3^ (Table 1) and it may be a founder variant. If half of the heterozygotes of this variant could trigger the lack of coagulation factor IX, the morbidity of Factor XI deficiency in the Chinese population would be far higher than the currently speculated (0.1–246.2 × 10^−6^) (10). In fact, Asselta et al. estimated that disease-causing *F11* heterozygote frequency was 0.0058 in the global population and 0.0090 in the East Asian population (20). This evidence suggests that the incidence of Factor XI deficiency may be severely underestimated. *F11* variant screening can help us understand the widespread presence of Factor XI deficiency and prevent related complications.

To gain insight into the characteristics of *F11* variants, we mapped 183 known missense variants (the data were from HGMD and literature sources; Figure 3A) (9, 20–26). Of these, 45.05% were located in the Protease domain, the four Apple domains shared 53.29% of the variants, and the other variants only occupied 1.65% in all variants (Figures 3A,B). After further analyzing the effects of these variants, more than half of the variants (53.33%), including our variant p.V611G, were found to change the hydrophobicity, and an electrical charge change (36.26%) and the disappearance or acquisition of a ring structure (39.56%) also were common pathogenic mechanisms. In addition, 12.09% of the variants broke disulfide bonds (Figures 3A,C). Our data can assist clinicians in the assessment of the pathogenicity of novel variants.

## Conclusion

In this study, we recruited a Chinese family with Factor XI deficiencies and identified two *F11* variants (NM_000128.4: c.841C>T, p.Q281X [known]; c.1832T>G, p.V611G [novel]) in the patients. Our report again revealed that heterozygous variants were responsible for mild Factor XI deficiency and biallelic variants caused severe type. Furthermore, we conducted an extensive review of the features of *F11* missense variants. Our findings not only expand the variant map of *F11* but also contribute to genetic counseling and molecular diagnostics in Factor XI deficiency.

## Data Availability

The original contributions presented in the study are included in the article/Supplementary Material, further inquiries can be directed to the corresponding author.
